# Flexible Temperature Sensor Array Based on a Graphite-Polydimethylsiloxane Composite

**DOI:** 10.3390/s100403597

**Published:** 2010-04-09

**Authors:** Wen-Pin Shih, Li-Chi Tsao, Chian-Wen Lee, Ming-Yuan Cheng, Chienliu Chang, Yao-Joe Yang, Kuang-Chao Fan

**Affiliations:** Department of Mechanical Engineering, National Taiwan University, Taipei, Taiwan; E-Mails: r94522525@ntu.edu.tw (L.-C.T.); r96522514@ntu.edu.tw (C.-W.L.); mingyuan@mems.me.ntu.edu.tw (M.-Y.C.); clchang6@ntu.edu.tw (C.C.); yjy@ntu.edu.tw (Y.-J.Y.); fan@ntu.edu.tw (K.-C.F.)

**Keywords:** composite, temperature sensor array, flexible substrate

## Abstract

This paper presents a novel method to fabricate temperature sensor arrays by dispensing a graphite-polydimethylsiloxane composite on flexible polyimide films. The fabricated temperature sensor array has 64 sensing cells in a 4 × 4 cm^2^ area. The sensor array can be used as humanoid artificial skin for sensation system of robots. Interdigitated copper electrodes were patterned on the flexible polyimide substrate for determining the resistivity change of the composites subjected to ambient temperature variations. Polydimethylsiloxane was used as the matrix. Composites of different graphite volume fractions for large dynamic range from 30 °C to 110 °C have been investigated. Our experiments showed that graphite powder provided the composite high temperature sensitivity. The fabricated temperature sensor array has been tested. The detected temperature contours are in good agreement with the shapes and magnitudes of different heat sources.

## 1. Introduction

Recently, intensive efforts have been taken to develop sensing systems for intelligent robots. One of the focuses on this subject is the design and fabrication of humanoid artificial skin. The successful implementation of artificial skin relies on the improvement of its sensitivity and mechanical flexibility. Most investigations of artificial skin emphasize the touch or tactile sensing to imitate the somatic sense of real human skin. Tajima *et al.* [1] developed a multi-layer touch sensor integrated with soft materials by microelectromechanical systems (MEMS) technology. Engel *et al.* [2] and Someya *et al.* [3] presented soft and flexible tactile sensor arrays for robotic applications. As a matter of fact, the sensory functions of human skin include not only the touch sensation but also pressure, temperature, pain, or other compound reactions from receptors and nerve endings. In the development of more sophisticated skin-like sensing systems, Siegel *et al.* [4] mounted 8 × 8 force sensing cells and 4 × 4 temperature sensors in a stacked rigid substrate, which was then implemented on a robot finger. Their temperature sensors are based on detecting heat loss due to ambient temperature variations. To develop flexible sensor array, Someya *et al.* [5] fabricated networks of pressure and thermal sensors using active matrices of organic transistors. Han *et al.* [6] employed platinum microresistors to form a one-dimensional flexible temperature sensor array. Temperature sensing systems have yet to become more commonplace in the field of robotic sensation. Besides Someya *et al.*’s work [5], reports about large arrays of temperature sensors on flexible substrate are still very rare.

The development of a flexible sensor array relies on an appropriate sensing mechanism and a suitable large-area fabrication technique. Our preliminary study showed that a polymer dielectric filled with conductive particles could be a good candidate to provide both mechanical flexibility and temperature sensitivity to the sensor array [7]. When the volume fraction of conductive particles inside a polymer dielectric approaches a percolation threshold, the electrical resistivity of the composite dramatically drops. This is because the number of the conductive particles reaches a critical value at which some “electrical pathways” are formed in the polymer matrix [8]. The associated material model is based on general effective media theory and has been intensively studied [9–12]. The electrical resistivity of the conductor-polymer composite could change significantly in response to mechanical stress or temperature variations. In theory, conductive fillers with suitable conductivity and aspect ratio can make the electrical resistivity of the composite sensitive to temperature changes [11]. Although the piezoresistivity of similar composites subjected to external stresses has been extensively studied [11,13–18], the temperature response and associated sensor development have not been reported. In this paper, we investigate the resistivity-temperature characteristics of a graphite-polydimethylsiloxane (PDMS) composite. A bending test is also conducted to verify the resistivity response of the composite. To fabricate a sensor array based on the investigated carbon-polymer composites, we demonstrate a mass production process which employs an automatic dispensing system that is used to apply the composites onto a flexible circuit board. The function of the fabricated flexible sensor array will be shown.

## 2. Materials

In this work, electro-resistive polymer is used as the temperature sensitive material. This material is made of polydimethylsiloxane (PDMS) with graphite powder which serves as the conductive filler. Carbon particles are commonly used as conducting fillers because of their high receivability, low cost, and simple fabrication. For fabricating sensor arrays using micromachining techniques, PDMS is commonly used as the polymer matrix for its simplicity in the fabrication process and its high chemical stability [19,20]. Although the temperature sensors will be fabricated on polyimide substrate which is commercially available, the control of residual stress in polyimide is still under investigations [21–24]. Therefore, polyimide is not used as the polymer matrix. The PDMS matrix is SYLGARD^®^ 184 from Dow Corning, and its specific gravity is 1.03 kg/m^3^ at 25 °C. The mixing ratio of curing agent and prepolymer is 1:10 in weight. The thermal conductivity and volume resistivity of the PDMS matrix are 0.18 W/m-K and 1.2 × 10^14^ Ω-cm, respectively. Hence the PDMS matrix is intrinsically a good heat and electric insulation material. The advantages of using PDMS as the insulation matrix also include its high durability, low fabrication temperature, and simple fabrication process.

**Figure 1.**
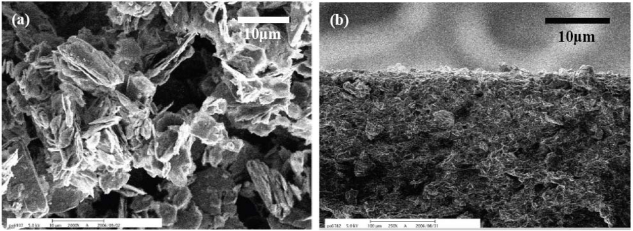
SEM images of (a) graphite powder and (b) a graphite-PDMS composite.

In order to make the insulation matrix conductive, we interfused conductive fillers such as carbon black (Ketjen Black EC300J), graphite powder (Sigma-Aldrich No.15553), carbon nano-fibers (Showa Denko VGCF^®^-H) into PDMS. The temperature sensitivity of the composites based on these three different conductive fillers was evaluated separately. The image taken with a scanning electron microscope (SEM) in Figure 1(a) indicates that the mean diameter of the graphite powder is about 10 μm and that the shape is irregular. It is worth mentioning that the electrical conductivity of polymer-graphite composites would increase with mean diameter of the graphite [25]. The cross-sectional SEM image of a graphite-PDMS composite is shown in Figure 1(b).

**Figure 2.**
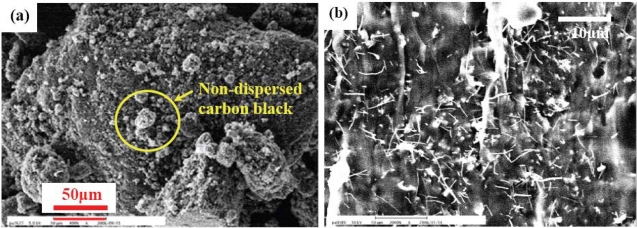
SEM images of (a) carbon black in PDMS and (b) carbon nano-fibers in PDMS.

Figure 2(a) shows the carbon black inside the PDMS matrix. These carbon black particles have the original diameter of 30–35 nm and aggregate easily without careful dispersion during mixing process. These aggregated carbon black particles induce electrical instability when the composites are subjected to temperature variations. Carbon nano-fibers were also mixed with PDMS for comparison, as shown in Figure 2(b). It was found that the resistivity of such composites barely changes with temperature variations. In such composites, large aspect ratio of carbon nano-fibers results in multi-conductive networks in insulating PDMS. Even though the connection of a few conducting paths would be broken during thermal expansion, most of the carbon nano-fiber network remains when the ambient temperature increases. Comparing to carbon black and carbon nano-fibers, we have found that graphite powder as the conductive-filler could provide much higher temperature sensitivity of the electro-resistive PDMS.

**Figure 3.**
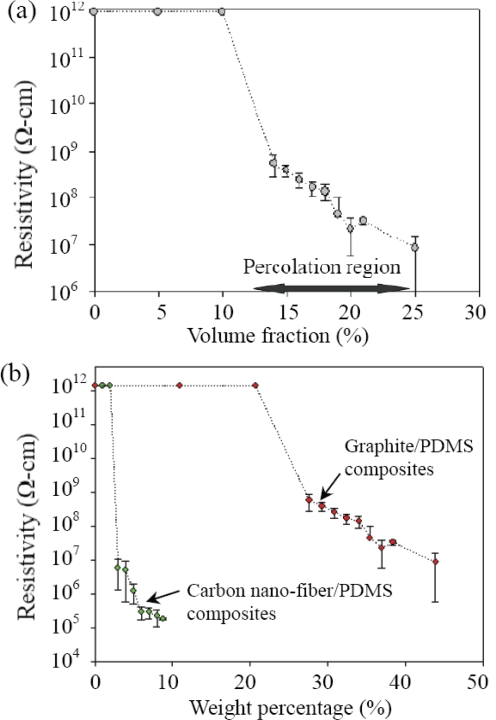
(a) Percolation threshold of graphite/PDMS composites. (b) Resistivity of graphite/PDMS and carbon nano-fiber/PDMS composites at different weight percentage of the conductive fillers.

We fabricated the composites of different volume fractions of the graphite powder in PDMS to determine the percolation threshold. The resistivity of these composites was measured, as given in Figure 3(a). For each volume fraction of the composite, we measured the resistivity of 25 samples. The error bar shows the maximum and the minimum value of the measurement.

For each volume concentration, the resistivity variation is mainly caused by the non-uniformity of the composite. Therefore, independent calibration is required for the composite to be used for sensor applications.

Figure 3(a) indicates that the percolation threshold occurs between 12% and 25% of the graphite volume fraction. The measured resistivity of the samples with 25% volume fraction shows wider range, which implied poorer uniformity of the composite. This is because the high viscosity of the graphire/PDMS mixture makes it difficult to disperse graphite powder uniformly. The resistivity of the composite could be further decreased if the volume fraction of graphite is greater than 25%. However, the composite becomes very difficult to dispense due to the high viscosity.

The resistivity of graphite/PDMS and carbon nano-fiber/PDMS composites can be compared in Figure 3(b). For materials in nanometer scale, it is more common to describe the amount by weight. Hence the concentration in Figure 3(b) is shown by the weight percentage of the conductive filler to the composite. Comparing to graphite, it requires less amount of carbon nano-fiber for the composite to reach percolation region. This is because the carbon nano-fiber has much higher aspect ratio.

## 3. Design and Fabrication

The flexible substrate of our temperature sensor array is comprised of copper electrodes, wires, and polyimide insulation layers. The copper interconnects are patterned with these layers to form a complete scanning circuitry. The details of the scanning circuit can be found in [26,27]. The interdigitated electrodes are designed upon the insulation layer, and the electro-resistive PDMS is applied on these electrodes by an automatic dispensing system. The resistance of the sensing material changes immediately with the ambient temperature variation, and signals can be detected by the scanning circuitry.

**Figure 4.**
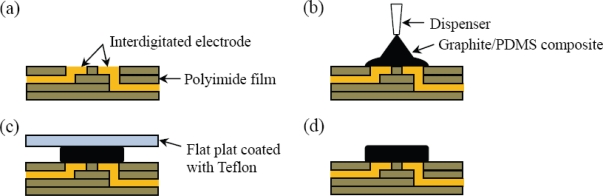
Process flow for fabricating the temperature sensor array. (a) Flexible printed circuit. (b) Applying graphite/PDMS composite on the interdigitated electrode. (c) Applying a flat plate coated with Teflon to flatten the composite. (d) Curing the composite and then removing the plate.

To fabricate the temperature sensor array, the graphite-PDMS composite must be prepared beforehand. The conductive filler, graphite powder, together with dispersant was blended into PDMS prepolymer at the first step. A dispersant such as cyclohexane increases the fluidity of the PDMS prepolymer and improves the mixture uniformity through the blending procedure. The blending procedure included ultrasonic mixing and stirring steps and was conducted for 30 minutes. Then the PDMS curing agent was well mixed into the composite. We degassed all the mixture in a vacuum chamber for 30 minutes to evaporate the volatile cyclohexane. In this step, we did not observe any migration of the graphite caused by the gravity. The process for applying the unpolymerized sensing material onto the flexible printed circuit is depicted in Figure 4. The composite was put into a dispensing machine to be selectively applied on the designated interdigitated copper electrodes, as shown in Figure 4(b). This automatic dispensing system is an SR-300D from Homytech Auto Part Co., Taiwan. It has a minimum motion step of 50 μm and is therefore capable of addressing high-resolution sensor arrays. By controlling the three-axis movement platform of the dispensing system, the graphite-PDMS composite can be well aligned with the pattern of the sensing electrodes. The transferring and dispensing steps took less than 10 minutes. Due to the high viscosity of the mixture, the uniformity of the volume fraction could not be affected. There are 64 sensing electrodes distributed over a 4 × 4 cm^2^ area, and the dimension of each interdigitated electrode is 1.5 × 1.5 mm^2^. Hence the volume of the graphite-PDMS composite on each sensing electrode could constantly be kept small.

**Figure 5.**
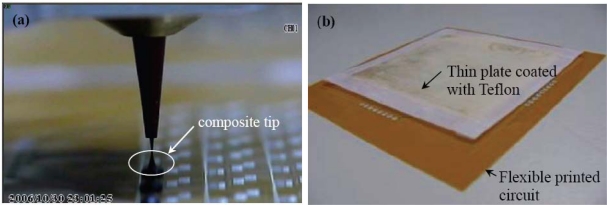
(a) The viscosity of the graphite-PDMS composite results in a sharp tip on each sensor cell during the dispensing process. (b) A thin flat plate coated with Teflon is used to flatten all sensor cells.

**Figure 6.**
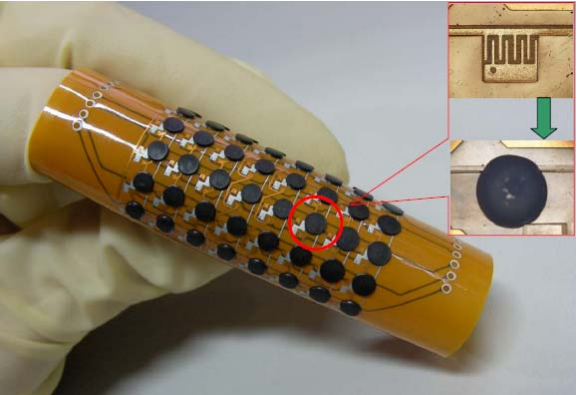
Fabricated flexible temperature sensor array. The insets show the interdigitated electrode and composites on the electrode, respectively.

The graphite-PDMS composite becomes more viscous as the volume fraction of the graphite powder increases. The volume fraction of the conductive fillers must be robust for adequate electrical resistance and manufacture feasibility. The graphite-PDMS is dispensed at room temperature to avoid the dispenser from clogging up over time. The PDMS curing time at room temperature is much longer than the time, which is about ten minutes, for dispensing the composite on 64 electrodes [28]. To overcome the high viscosity of the graphite-PDMS composite, a constant pressure of 294 kPa is applied to dispense the composite. When the automatic dispensing system moves up and down, it will cause sharp tips of residue PDMS as shown in Figure 4 (c) and Figure 5 (a). The composite sticks to the copper and the substrate very well without any modifications on the polyimide surface. These sharp tips will cause non-uniform contact between heat source and every single sensor cells, resulting in inaccurate temperature contour of the sensor array in practical implementation. To eliminate these sharp tips, we pressed the sensor array with a thin flat plate, on which the surface energy has been reduced by Teflon coating, as shown in Figure 4(c) and Figure 5(b). Then we put the sensor array on a hotplate and cured the PDMS at 80 °C for 8 hours to completely evaporate the cyclohexane solvent. Finally, we removed the flat plate, as shown in Figure 4(d). The fabricated temperature sensor array exhibits good flexibility, as shown in Figure 6. The insets show the sensing electrode and an electrode covered with the composite, respectively.

## 4. Experiments and Discussion

**Figure 7.**
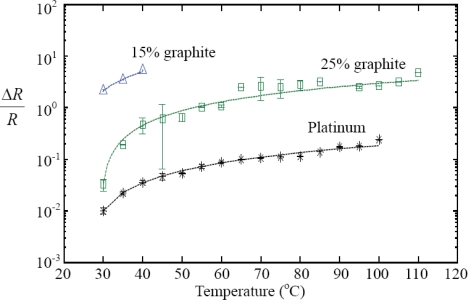
Measured resistance change of the graphite-PDMS composites subjected to different temperature values. The volume fractions of the graphite powder in the composites are 15% and 25%, respectively.

PDMS with three different volume fractions of graphite powder (15% and 25%) were chosen to determine the resistance variations by altering temperature on a hotplate in an air atmosphere. These volume fractions were chosen for higher temperature sensitivity as they are in the range of the percolation threshold. After degassing and curing, the sheets of the graphite-PDMS composites were cut into 1 × 1 cm^2^ pieces and then sandwiched by copper plates. The thickness of these PDMS sheets was fixed at 0.5 mm by using a scraper before polymerization. The copper plates were connected to a multimeter for resistance measurement while the temperature on the composite sheets was increased from 25 °C to 110 °C on the hotplate. Before each measurement, the temperature was increased by 5 °C and then maintained as a constant for 10 minutes to assure thermal equilibrium between testing sample and hotplate. The relative humidity of the testing environment was maintained at 60%. Figure 7 shows the measured resistance variation of the graphite-PDMS composites subjected to different temperature. Platinum thin film as a temperature sensor was also characterized for comparison. The temperature coefficient of resistance (TCR) can be calculated by:

(1) $$\alpha =\frac{\Delta R/R_{0}}{T-T_{0}}$$

where *T_0_* is the ambient temperature, and *R_0_* is the initial resistance of tested sample. Equation (1) is used to extract the TCRs in our experiment. The fitting curves are shown as the dashed lines in Figure 7.

The experimental results agree with the linear model. According to the data, the platinum TCR is determined to be 0.0055 K^−1^. The TCRs of the composites are 0.042 K^−1^ and 0.286 K^−1^ for the graphite volume fractions of 25% and 15%, respectively. The testing results indicate that the sensitivity of the graphite-PDMS composites is higher than that of typical platinum temperature sensors [29]. Although the uniformity of the graphite-PDMS composites still has to be improved, these composites have been demonstrated to have potential applications in automatic or robotic systems. Prominent increase of resistance in these composites is also observed as the volume fraction of graphite powder decreases. However, the sensation range also drops with decreasing volume fraction. For the 15% volume fraction, the temperature sensing polymer becomes insulating when the temperature is greater than 40 °C. The thermal expansion of the PDMS could break contact between graphite particles in the composite [30]. Because the sample of 15% volume fraction is close to the lower bound of the percolation region as shown in Figure 3(a), all the conductive paths in the composite are broken at over 40 °C. Therefore, this temperature sensing polymer is suitable to be a switch apparatus for protecting objects from high temperature sources because of its sharp increase in resistance. The experimental result in Figure 7 implies that the 25% volume fraction composite has lower TCR than the 15% volume fraction composite. More rigorous experiment is required for fully understanding the TCRs of this composite in the future.

**Figure 8.**
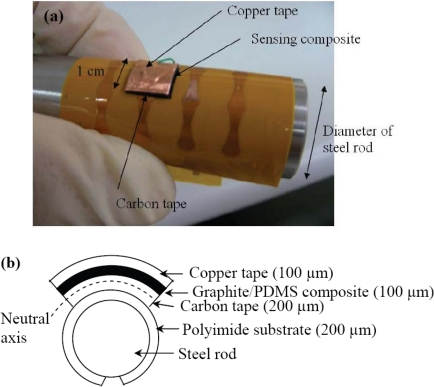
The sensing composite in 1 cm^2^ area was sandwiched between a conductive copper tape and a conductive carbon tape on a flexible printed circuit board. Its resistance was then measured on steel rods of different diameters. (a) Photograph of the bending test. (b) Side-view illustration.

The flexibility of the fabricated sensing composite with 20% volume fraction of graphite powder has been characterized. In this test, the sensing composite in 1 cm^2^ area was sandwiched by a conductive copper tape and a conductive carbon tape on the flexible printed circuit board, as shown in Figure 8.

**Figure 9.**
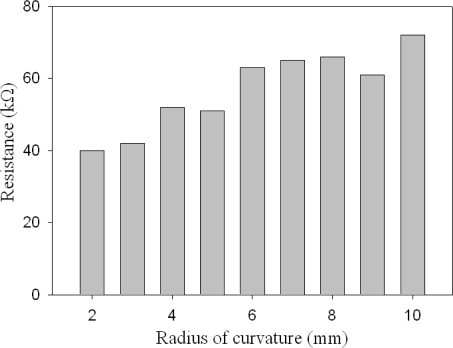
The measured resistance of the sensing composite increases with increasing bending radius of curvature.

The configuration in Figure 8(b) is to shift the material away from the neutral axis so that the material can be completely under tensile stress. The sensing composite was then wrapped around steel rods, and its resistance change was measured. The steel rods have different curvature radii from 2 mm to 10 mm with 1 mm increment. The testing results in Figure 9 indicate that the resistance decreases with the radius of curvature. This is because the composite was put at the convex side in the test and hence was subjected to tensile stress. The tensile stress stretches the composite laterally, causing the thickness to decrease. Thinner composite has smaller resistance. This bending test implies that the sensing signal of the composite could be affected by external stress. To remove the bending effect, one can simply put the composite close to the neutral axis by change the thickness ratio of the material to the substrate.

**Figure 10.**
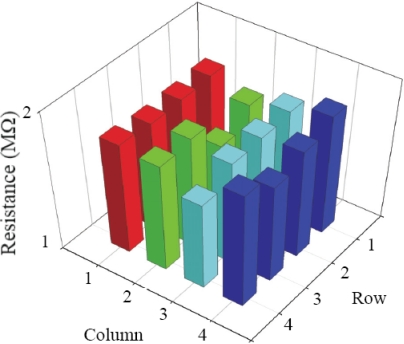
A 4 × 4 resistance map of the fabricated sensor array.

The fabricated temperature sensor array of 20% volume fraction of graphite powder was connected to the scanning circuits for full-function evaluation. At room temperature, the resistance varies randomly between 1.43 MΩ and 1.76 MΩ across the sensor array. For easy observation, a 4 × 4 resistance map of the array is shown in Figure 10.

**Figure 11.**
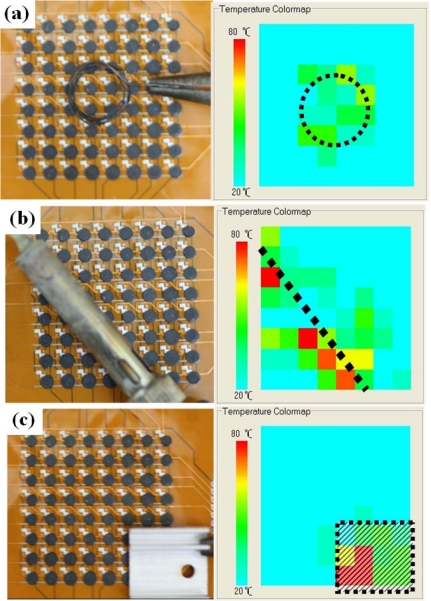
Measured temperature distributions of the fabricated sensor array subjected to different heat sources. (a) Heated ring. (b) Heated rod. (c) Heated rectangular plate.

The resistance variation could be due to the thickness and shape non-uniformity since they were controlled by applying the thin plate for flattening all sensor cells. Nevertheless, measurement uniformity could be achieved by conducting independent calibration of each sensor cell. We also improved the performance uniformity of the sensor array by adjusting scanning circuit program. Then heat sources with different shapes were applied 5 mm above the temperature sensor array. The heated ring is made of iron wire. The heat rod is soldering iron. The heated plate is made of bulk aluminum. The temperature increase is successfully detected by each sensor cell, and the overall temperature is displayed by the contour map shown in Figure 11. These contours are in well agreement with the shapes and temperature scales of the applied heat sources. The non-uniform temperature distribution could be caused by the heat source, background noise, or ambient fluctuations.

## 5. Conclusions

A flexible temperature sensor array consists of a graphite-PDMS composite, metal sensing electrodes, and flexible polyimide film has been successfully fabricated. There are 8 × 8 sensor cells in a 4 × 4 cm^2^ area with good flexibility and robustness. Our experiment shows that polymer mixed with conductive fillers exhibits resistance variation when it is subjected to ambient temperature change. Graphite powders mixed with PDMS is found to have the highest temperature sensitivity and higher stability compared with the composites using other carbon fillers. As the volume fraction of graphite powders reaches the percolation threshold, the resistivity of the composites drops substantially. Different volume fractions of graphite powders have been investigated. The composite with 15% graphite powder are suitable for on/off devices while the one with 20% graphite powder provides sufficient dynamic range for continuously sensing temperature changes. The function of the fabricated temperature array has been verified through a resistance scanning system. The detected temperature contours agree well with the shape and magnitude of the applied heat source. An automatic dispensing method has been developed to fabricate this temperature sensor array. Larger sensor array can be easily achieved using the same process for robotic applications.
